# Mathematical Modeling and Simulation of Adaptive Nozzle Design in Material Extrusion

**DOI:** 10.3390/ma18214954

**Published:** 2025-10-30

**Authors:** Donghui Kim, Seong Je Park, Seung Ki Moon

**Affiliations:** 1 School of Mechanical and Aerospace Engineering, Nanyang Technological University, 50 Nanyang Avenue, Singapore 639798, Singapore; donghuikim@gmail.com; 2 Singapore Centre for 3D Printing, School of Mechanical and Aerospace Engineering, Nanyang Technological University, 50 Nanyang Avenue, Singapore 639798, Singapore; 3 School of Mechanical Engineering, Gyeongsang National University, 501 Jinju-daero, Jinju-si 52828, Gyeongsangnam-do, Republic of Korea

**Keywords:** additive manufacturing, adaptive nozzle, finite element analysis, mathematical modeling

## Abstract

This study proposes an adaptive nozzle design for material extrusion-based food additive manufacturing (AM), integrating both mathematical modeling and finite element analysis. A theoretical framework is developed to correlate extrusion radius and nozzle diameter with process parameters such as feeding speed, nozzle velocity, and shear rate. The proposed model is extended to estimate volumetric extrusion rate and incorporate rheological parameters using the Hagen–Poiseuille relation. To validate the derived equations, static structural simulations are conducted in a computer simulation under varying pressures, nozzle diameters, temperatures, and input feeding diameters. The simulation results show that increased pressure and higher temperatures enhance extrusion efficiency, while larger nozzles and feeding diameters reduce flow resistance and improve extrusion stability. Collectively, these findings validate the predictive capability of the mathematical model and highlight the feasibility of adaptive nozzle systems for optimizing extrusion performance in food AM. The study provides a preliminary foundation for the future development of dynamic nozzle control strategies that enable improved print fidelity and process flexibility.

## 1. Introduction

Additive manufacturing (AM) has evolved into a key technology for producing customized and complex geometries across a wide range of engineering fields [1]. According to the ISO/ASTM 52900 standard [2] AM can be classified into seven groups depending on the deposition mechanism and the type of materials used: binder jetting, directed energy deposition, material extrusion, material jetting, powder bed fusion, sheet lamination, and vat photopolymerization [3]. Each process offers distinct advantages, but among them, material extrusion (MEX) has received particular attention due to its simplicity, cost-effectiveness, and versatility [4]. MEX is widely adopted in prototyping and in end-use part production. And it allows precise control over deposition parameters while maintaining relatively low equipment and operational costs. In addition to common thermoplastic filaments such as polylactic acid (PLA), acrylonitrile butadiene styrene (ABS), polyamide (PA), and polyetheretherketone (PEEK), MEX can process composite and fiber-reinforced filaments, as well as paste-like materials, including concrete, chocolate, and dough [5,6,7,8]. This broad material compatibility has extended its applications across mechanical, biomedical, mold, and construction industries [9,10].

The advantages of MEX have also led to significant progress in food AM. For instance, recent studies have demonstrated the feasibility of fabricating multi-material 3D-printed foods using diverse edible pastes such as corn dough and chickpea paste, followed by post-processing steps like steaming or baking to adjust appearance, weight, dimensions, and texture. It was reported that steamed samples exhibited fewer surface cracks and improved visual quality compared to oven-cooked ones [11]. In addition, the influence of post-AM treatments on dimensional stability and textural properties has been highlighted. Cooking methods were found to significantly affect moisture retention, shrinkage rate, and mechanical strength, demonstrating that processing conditions are critical to ensuring the quality of printed foods [11]. Beyond structural fidelity, MEX-based food AM has also contributed to the field of personalized nutrition. For example, the incorporation of hydrocolloids has been shown to enhance both printability and viscoelastic properties, enabling the production of soft-textured foods tailored to elderly populations [12]. Furthermore, process monitoring technologies of food have advanced in parallel. Computer-vision-based methods have been introduced to quantify extrusion rate and line width in real time, allowing improved control of deposition quality even under constant extrusion pressure or force [13]. While efforts have significantly advanced MEX-based food AM, another crucial challenge arises from the need to accommodate food materials with widely varying viscosities during extrusion.

When it comes to AM highly viscous food materials, the nozzle diameter plays a crucial role. A larger nozzle is typically required to reduce flow resistance and prevent excessive pressure build-up, whereas for low-viscosity materials, a smaller nozzle can be sufficient to ensure precision in deposition [14]. This relationship arises because extrusion pressure must overcome both material viscosity and nozzle-induced shear stress, meaning that the same nozzle diameter can behave very differently depending on the rheological properties of the feedstock [15]. Furthermore, previous studies have shown that limited research has systematically examined the correlation between rheological parameters and extrusion performance, which restricts the predictability and stability of AM outcomes [16]. It has also been observed that food inks with excessively high viscosity, typically in the range of approximately 100,000 to 200,000 cps, tend to cause backflow and unstable extrusion, leading to a loss of geometric fidelity AM [17]. These findings collectively indicate the need for adaptive extrusion systems capable of accommodating diverse material properties, an aspect that is addressed in the present study through the development and simulation of an adaptive nozzle.

In this study, we propose the development of an adaptive nozzle system capable of dynamically adjusting its diameter in response to real-time AM conditions. A mathematical model is formulated to govern the behavior of the adaptive nozzle, incorporating key rheological parameters such as shear rate, viscosity, extrusion rate, and nozzle radius. Furthermore, in order to provide a preliminary validation of the proposed mathematical model, finite element analysis (FEA) is performed using static structural simulations. The FEA assesses the mechanical performance, stress distribution, and extruding behavior of the adaptive nozzle under varying extrusion conditions such as pressure and temperature. The results provide critical insights into the feasibility and optimization of adaptive nozzle technology, ensuring its applicability in next-generation food AM.

## 2. Methodology

### 2.1. Mathematical Model Development

**Figure 1:**
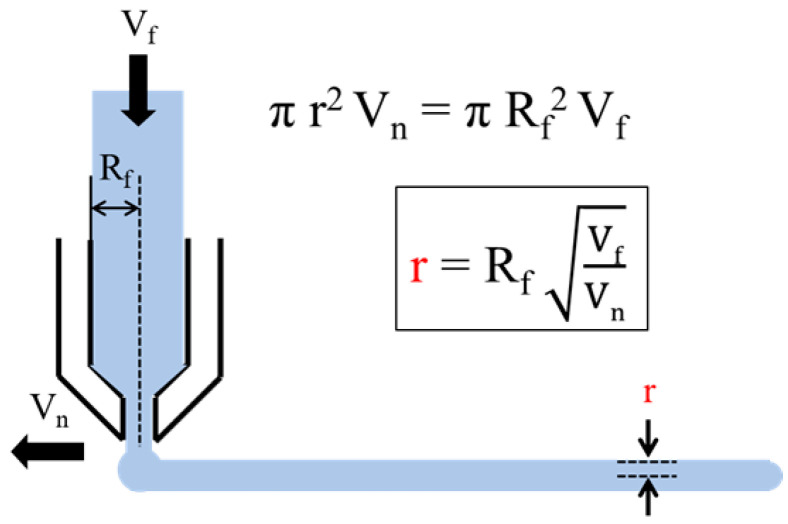
Relationship between process parameters and the extruded radius (r) in MEX [18].

A mathematical framework was established to describe the extrusion behavior in material extrusion MEX-based food AM. The model aimed to link the filament radius and nozzle diameter with key process parameters such as feeding speed, nozzle velocity, and shear rate. Starting from mass conservation principles, the extruded radius (r) was defined as a function of feeding speed (V_f_) and nozzle speed (V_n_) as shown in Figure 1 [18]. The governing equations were further extended to incorporate volumetric extrusion rate and rheological parameters, including shear rate derived from the Hagen–Poiseuille relation. These derivations provided a predictive basis for determining optimal nozzle diameters under varying conditions.

### 2.2. Computational Simulation

**Figure 2:**
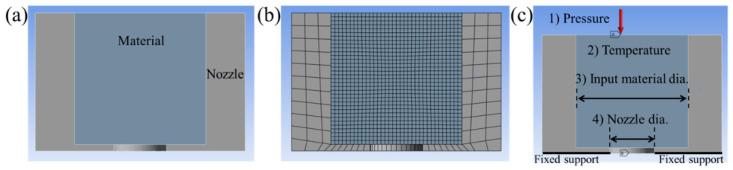
FEA setup for nozzle extrusion: (**a**) cross-section of the nozzle geometrical model, (**b**) meshing with refined elements in the materials zone, and (**c**) boundary conditions.

FEA was conducted using the static structural analysis module in ANSYS Workbench 2023 to validate the derived equations under various operating conditions. The geometrical model of the nozzle is presented in Figure 2a, where a cylindrical channel was constructed to represent the extrusion path. As shown in Figure 2b, the mesh is designed to be dense with a mesh size of 0.3 mm with quad/hex in the material zone because the deformation of the material is important. The mesh of the nozzle is roughly designed as a default with a quad/hex. Figure 2c illustrates the applied boundary conditions, including four variables: pressure (1.5, 3.0, 4.5, 6.0, and 7.5 kPa), temperature (room temperature, 50, 70, and 90 °C), input material diameter (10, 11, 12, 13, 14 mm), and nozzle diameter (2, 4, 8 mm). The parameter values for pressure, temperature, input material diameter, and nozzle diameter were determined through preliminary simulations aimed at identifying representative conditions that ensure numerical stability and observable deformation trends within the feasible extrusion range. In addition, the bottom surface of the nozzle was constrained with a fixed support, and a symmetry boundary condition was imposed on the cross-sectional plane. The structural steel and polyethylene were selected as the nozzle and material, respectively.

## 3. Results and Discussion

### 3.1. Mathematical Model Analysis

In MEX-based food AM, a mathematical model is required to quantitatively control the user-tailored texture as well as the nutritional content and flavor of the extruded material. Park et al. described a mathematical model to predict the radius of extruded material in a MEX-based process, as shown in Figure 1 [18]. The model describes the relationship between the radius of the extruded material and key process parameters, expressed as r = R_f_ (V_f_/V_n_) − 1, where r is the radius of the extruded material, R is the radius of the input material, V_f_ is the feeding speed of the input material, and V_n_ is the movement speed of the nozzle as shown in Figure 1. However, in food AM, it is essential not only to predict the radius of the extruded material but also to calculate the final volume of the output and the precise amount of ingredients used in the layering process. This is crucial for creating user-tailored products, as factors such as texture, density, nutritional content, and flavor directly impact the quality of the Food AM output. Therefore, to effectively control these aspects, it is necessary to expand the model proposed by Park et al. to include calculations for volume and material usage.

To calculate the amount of ingredients used in food AM, it is necessary to determine the volume of the extruded material based on the radius and the path of the nozzle movement. For instance, the volume V of material extruded in a single layer can be calculated using the formula V=πr^2^⋅L, where r is the radius of the extruded material and L is the nozzle’s movement path or the length of the deposited material. This formula is useful for calculating the amount of ingredients used in a single layer, and it can be applied iteratively to estimate the total amount of ingredients used across all layers. As the layers accumulate, the total volume of the final output can be expressed as the sum of the volumes of individual layers: V_total_= $\sum_{i=1}^{n}\pi r_{i}^{2}\cdot L_{i}$ Here, n represents the total number of layers in the output, while r_i_ and L_i_ denote the radius and movement path of the extruded material for each specific layer. This calculation approach is critical for accurately predicting the amount of dough required to produce specific food items, minimizing material waste, and maintaining the quality of the final product.

While Park et al.’s model provides a fundamental framework for predicting the radius of the extruded material, one notable limitation is that the equation does not explicitly incorporate the nozzle diameter as a factor. To compensate for this limitation, Park et al. determined an appropriate nozzle diameter experimentally, optimizing the extrusion conditions through trial and error rather than integrating it as a predictive factor in their mathematical model. A nozzle’s diameter significantly affects the precision and surface texture of AMed objects [19,20]. A larger nozzle diameter results in coarser structures with increased surface roughness, while a smaller nozzle diameter produces more delicate and detailed structures [21]. Both the extrusion rate and nozzle movement speed simultaneously influence AM quality, as they determine the amount of material extruded per unit length and time. This study investigates the relationship between high and low extrusion rates in comparison to the targeted product quality. The objective is to determine the optimal extrusion rate for achieving a smooth, uniform line with a consistent diameter. The proposed equation calculates the extruded volume rate (mm^3^/s) given by Equation (1). V_n_ is the movement speed of the nozzle, and *D*_n_ is the nozzle diameter. By assuming that V_n_ in Figure 1 is equivalent to V_n_ in Equations (1) and (2) can be derived [18].

V_d_ = (π/4)·V_n_·*D*_n_^2^ = (π/4) (1)

(2) $$D_{n}=\frac{2r}{R}\sqrt{\frac{V_{d}}{\pi V_{f}}}$$

To incorporate rheological behavior into the nozzle diameter equation, the volumetric extrusion rate V_d_ in Equation (2) was expressed in terms of shear rate. From the Hagen–Poiseuille relation [22], the shear rate is defined as

(3) $$\dot{\gamma }=\;4Q/\pi R^{3}$$

where Q is the volumetric flow rate and R is the inner radius of the feeding material. By rearranging Equation (3), the flow rate can be written as [22]:

(4) $$Q\;=\;\dot{\gamma }\pi R^{3}/4$$

Assuming V_d_ ≈ Q, substituting Equation (4) into Equation (2) yields [18,22]

(5) $$D_{n}=\frac{2r}{R}\sqrt{\frac{V_{d}}{\pi V_{f}}}=\frac{2r}{R}\sqrt{\frac{\dot{\gamma }\pi R^{3}}{4\pi V_{f}}}$$

After simplification, the final governing equation for nozzle diameter is obtained as:

(6) $$D_{n}=r\sqrt{\frac{\dot{\gamma }R}{V_{f}}}$$

This relation highlights the direct coupling between shear rate, feeding speed, and nozzle diameter in controlling extrusion quality. Especially, in MEX-based food AM, the rheological behavior of the AMed material plays a dominant role in determining the final dimensional stability of the shape [16,23]. As the viscosity decreases with increasing temperature or shear rate, the material becomes more deformable, leading to smoother deposition lines but also a greater risk of shape distortion after deposition. Conversely, excessively high viscosity can resist flow and cause discontinuous extrusion, resulting in irregular layer geometry [24]. Therefore, Equation (6) describes both the mechanical relationship among nozzle diameter, shear rate, and feeding speed and the balance between flowability and shape retention during AM. This coupling between rheological behavior and geometrical accuracy should be carefully optimized in adaptive nozzle design for food AM.

### 3.2. Numerical Validation Through FEA

The finite element analysis (FEA) was conducted to validate the derived equations and examine extrusion behavior under different process conditions. The results highlight the influence of pressure, nozzle geometry, material temperature, and input feeding diameter on extrusion performance. Although the finite element analysis in this study was conducted using a static structural approach rather than a fluid-dynamic model, each simulation parameter can be conceptually related to the governing variables in Equation (6). Specifically, the input material diameter and nozzle diameter correspond directly to R and D_n_ in the mathematical model. The applied pressure represents the feeding speed, V_f_, as a higher pressure increases the material’s flow rate during extrusion. Likewise, temperature variation influences the elastic modulus of the material, which is analogous to changes in shear rate, 𝛾˙, in flow analysis. Therefore, the static simulations provide a structural validation framework that is physically consistent with the flow-based mathematical formulation.

#### 3.2.1. Effect of Pressure and Nozzle Diameter

**Figure 3:**
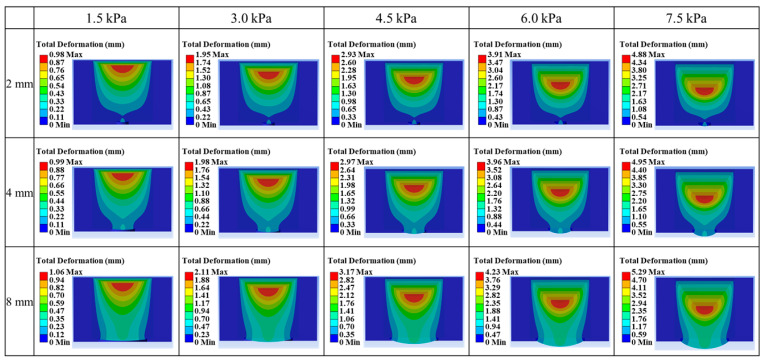
FEA results of extrusion behavior according to inlet pressure and nozzle diameter.

Figure 3 illustrates the combined effect of inlet pressure and nozzle diameter on extrusion behavior. The results reveal a clear threshold phenomenon: at small nozzle diameters (e.g., 2 mm), extrusion occurs only at the maximum applied pressure of 7.5 kPa, indicating that the driving force at lower pressures is insufficient to overcome the high flow resistance. In contrast, larger nozzle diameters (e.g., 8 mm) substantially reduce resistance, enabling extrusion under nearly all conditions except the lowest pressure of 1.5 kPa. These findings highlight the coupled role of pressure and nozzle geometry, consistent with theoretical models predicting that extrusion pressure must scale inversely with nozzle diameter [25]. Furthermore, the deformation behavior can be clearly interpreted from the red regions inside the material, which represent the areas of maximum deformation in each simulation. The extent of these red zones visually demonstrates how extrusion responds to variations in pressure and nozzle diameter. Quantitative observation indicates that total deformation increases almost linearly with applied pressure. For example, at 2 mm, deformation rose from approximately 1.0 mm at 1.5 kPa to approximately 4.9 mm at 7.5 kPa, confirming that extrusion is pressure-dominant. Additionally, deformation increases with larger nozzle diameters due to reduced flow resistance. This validates the consistency of the proposed mathematical framework with numerical observations.

#### 3.2.2. Effect of Temperature

**Figure 4:**
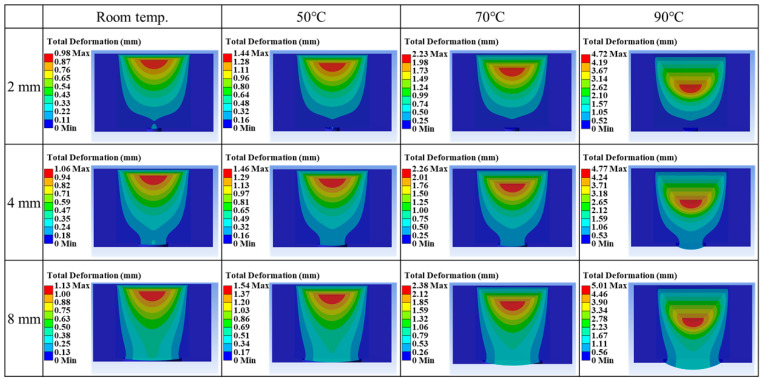
FEA results of extrusion behavior according to material temperature and nozzle diameter.

Figure 4 presents the effect of material temperature on extrusion when the inlet pressure is fixed at 1.5 kPa. Across all nozzle diameters, higher temperatures consistently improve extrusion efficiency. This behavior is attributed to the reduction of elastic modulus (or viscosity) with increasing temperature, which lowers flow resistance and facilitates material deformation within the nozzle. Furthermore, the red regions show that higher temperatures promote greater material flow. At 2 mm, 4 mm, and 8 mm nozzle diameters, the maximum deformation increased from room temperature to 90°C, showing nearly a 4.8-, 4.5-, and 4.4-fold enhancement in extrusion displacement, respectively. These results provide numerical evidence that the derived equations align with thermo-rheological principles, as elastic modulus (or viscosity) is known to decrease with temperature [26,27].

#### 3.2.3. Effect of Input Material Diameter

**Figure 5:**
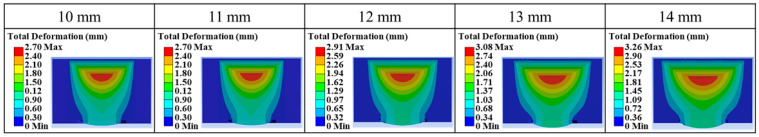
FEA results of extrusion behavior according to input material diameter.

Figure 5 shows the role of input material diameter while keeping the nozzle diameter, temperature, and inlet pressure constant at 8 mm, 90°C, and 7.5 kPa, respectively. The FEA results show that increasing the feeding diameter significantly enhances extrusion efficiency by reducing concentrations of local stress and increasing the material volume supplied to the nozzle. As shown in Figure 5, as the input material diameter increases from 10 mm to 14 mm, the red regions become slightly down, demonstrating that a larger feeding diameter promotes higher extrusion efficiency. For example, the maximum deformation increased from 2.7 mm at 10 mm input material diameter to 2.9 mm at 12 mm input material diameter, and then increased to around 3.3 mm at 14 mm input material diameter. This indicates that the extrusion performance improves with a larger input material diameter because of the lower concentration of local stress near the nozzle entrance. This finding underscores that extrusion performance is not solely governed by the outlet nozzle geometry, but also by the upstream feeding configuration. Therefore, a dual-adjustable nozzle design with independent control of both input feeding and output nozzle diameters is proposed as a potential strategy to improve extrusion performance and provide greater flexibility in tuning food quality.

Collectively, the results from Figure 3, Figure 4 and Figure 5 provide a comprehensive understanding of how extrusion is influenced by pressure, nozzle geometry, temperature, and input material diameter. The observed trends validate the predictive capacity of the derived equations, reinforcing their applicability to extrusion-based food AM. However, the static structural simulations employed here cannot capture the full rheological complexity of food materials, such as shear-thinning or viscoelastic behavior.

## 4. Conclusions

This study presented a combined mathematical and numerical investigation of an adaptive nozzle design for MEX-based AM. The mathematical model established a predictive relation between nozzle diameter and process parameters, while the FEA simulations validated the proposed mathematical model. The results demonstrated that extrusion performance was strongly influenced by pressure, nozzle diameter, material temperature, and input feeding diameter. Specifically, higher pressures and elevated temperatures enhanced extrusion efficiency, while larger nozzles and feeding diameters reduced flow resistance and improved the stability of the extrudate. These findings collectively support the feasibility of adaptive nozzle design as a strategy to improve extrusion-based food AM. However, the static structural simulations employed cannot capture the full rheological complexity of food materials, such as shear-thinning or viscoelastic behavior. For future research, advanced fluid dynamics simulations should be coupled with experimental validation to establish a correlation between viscosity, shear rate, and extrusion performance. Furthermore, the adaptive nozzle concept will be extended to a broader range of material systems, including polymers, ceramics, and composites, as well as to multi-material extrusion conditions. This expansion will allow the evaluation of adaptive nozzle performance across different geometries and application domains. Thus, the proposed concept for the adaptive nozzle will improve the predictive reliability of the model and enable optimized process design for practical food AM applications.

## Data Availability

The data presented in this study are available on request from the corresponding author. The data are not publicly available due to ongoing related research.
